# Preoperative Assessment of the Oral Environment in Patients Undergoing Tonsillectomy

**DOI:** 10.7759/cureus.100782

**Published:** 2026-01-04

**Authors:** Takafumi Yamano, Kensuke Nishi, Kensuke Kawamoto, Ryusei Nuita, Sayaka Sugimoto, Fumitaka Omori

**Affiliations:** 1 Section of Otorhinolaryngology, Department of Medicine, Fukuoka Dental College, Fukuoka, JPN; 2 Department of Dental Hygiene, Fukuoka Dental College Hospital, Fukuoka, JPN; 3 Department of Otorhinolaryngology, Fukuoka Dental College Hospital, Fukuoka, JPN

**Keywords:** chronic tonsillitis, oral cavity environment, the oral health assessment tool, tonsillar hypertrophy, tonsillectomy

## Abstract

Objective: Although maintaining oral hygiene may help prevent tonsillitis, the association between the two remains unclear. Tonsillectomy is a commonly performed procedure in otorhinolaryngology and has been studied from various perspectives; however, no studies have evaluated its relationship with the oral environment. We evaluated the influence of the oral environment on the pathogenesis of tonsillitis by comparing the preoperative oral environment in patients undergoing tonsillectomy according to surgical indication in a retrospective study.

Methods: We included 123 patients (64 male patients, 59 female patients) who underwent palatine tonsillectomy between April 2020 and March 2025. The mean age of the participants was 25.6 (4-81) years. Surgical indications were categorized into four groups for comparison: recurrent tonsillitis (N=54), peritonsillar abscess (N=23), tonsillar hypertrophy (N=36), and focal infection (N=10). Oral conditions were assessed within one month prior to surgery by a dedicated dental hygienist using the Oral Health Assessment Tool (OHAT).

Results: Significant differences were observed between the habitual tonsillitis and the tonsillar hypertrophy groups in the total OHAT score and the oral hygiene components. No significant differences were observed between the tonsillar hypertrophy and focal infection groups, the tonsillar hypertrophy and peritonsillar abscess groups, the focal infection and peritonsillar abscess groups, the focal infection and habitual tonsillitis groups, or the peritonsillar abscess and habitual tonsillitis groups.

Conclusion: The low OHAT score in the tonsillar hypertrophy group may be attributable to the higher proportion of pediatric patients in this group compared to the others. Future studies should incorporate more detailed oral hygiene measures and microbiological analyses to evaluate the relationship between tonsillar disease and the oral environment.

## Introduction

Improving the oral environment may contribute to the prevention of tonsillitis. Previous reports have indicated that the oral microbial environment is associated with tonsillar hypertrophy and chronic tonsillitis [1], improved oral hygiene may prevent tonsillitis [2], and disruption of oral microbiota may trigger inflammatory diseases [3]. However, the relationship between these factors remains insufficiently elucidated. At Fukuoka Dental College Hospital, all patients scheduled for surgery, including those undergoing palatine tonsillectomy, undergo a preadmission oral hygiene assessment. Tonsillectomy is a frequently performed procedure in otorhinolaryngology and has been investigated from multiple perspectives; however, the oral environment has not been evaluated in relation to surgical indication. We evaluated the influence of the oral environment on the pathogenesis of tonsillitis by comparing the preoperative oral environment in patients undergoing tonsillectomy according to surgical indication.

## Materials and methods

Subjects

We included 123 patients (64 male patients and 59 female patients; mean age 25.6 (4-81) years) who underwent palatine tonsillectomy at Fukuoka Dental College Hospital between April 2020 and March 2025 in a retrospective study. Patients were classified into four groups according to surgical indication, and intergroup comparisons were performed. The study population comprised all cases undergoing palatine tonsillectomy at our hospital during the observation period; no cases were excluded.

The habitual tonsillitis group comprised patients with a history of ≥ 3 episodes of acute tonsillitis within one year. The peritonsillar abscess group included patients with a history of ≥ 1 episode of peritonsillar abscess. The tonsillar hypertrophy group consisted of patients with upper airway obstruction due to enlarged palatine tonsils, presenting with obstructive sleep disorders, such as snoring or apnea. The focal infection group included patients in whom the palatine tonsils were considered a chronic source of inflammation and a potential focus for systemic disease.

Method of oral environment assessment

At the preoperative examination visit, approximately one month before surgery, the oral environment was assessed by a dedicated dental hygienist using the Oral Health Assessment Tool (OHAT). The OHAT is a screening instrument developed by Chalmers et al. [4] that is used by nursing and care staff to readily identify oral problems in individuals with disabilities and those requiring care.

The assessment comprised eight items: lips, tongue, gums/mucosa, saliva, remaining teeth, dentures, oral hygiene, and toothache. Each item was evaluated on a 3-point scale: healthy (0 points), changes (1 point), and unhealthy (2 points), with higher scores indicating a poorer oral environment.

Statistical analysis

The Kruskal-Wallis test was used for intergroup comparisons. For items showing statistically significant differences, post hoc multiple comparison tests were performed. Statistical analyses were performed using IBM SPSS Statistics for Windows, Version 29 (Released 2023; IBM Corp., Armonk, New York, United States).

This study was reviewed and approved by the Institutional Review Board of The Fukuoka Gakuen Research Ethics Committee (Approval No. 740). All procedures were conducted in accordance with the ethical standards of the Declaration of Helsinki and relevant guidelines and regulations.

## Results

Disease background

In total, 123 patients were classified into four groups according to surgical indications. The most common was the habitual tonsillitis group (54 patients: 20 male patients and 34 female patients; mean age 26: 8-49 years), followed by the tonsillar hypertrophy group (36 patients: 25 male patients and 11 female patients; mean age 15: 4-75 years), the peritonsillar abscess group (23 patients: 12 male patients and 11 female patients; mean age 37: 17-81 years), and the focal infection group (10 patients: four male patients and six female patients; mean age 36: 15-67 years). The focal infection group comprised six patients with palmoplantar pustulosis and four patients with IgA nephropathy. Compared to the other groups, the tonsillar hypertrophy group was skewed towards younger ages. Table 1 shows the mean values of the OHAT for the four groups.

**Table 1:** Mean Values of the OHAT for the Four Groups Total OHAT scores and item-specific scores categorized by the four surgical indications. OHAT: Oral Health Assessment Tool

	Total Score	Lips	Tongue	Gums/mucosa	Saliva	Remaining Teeth	Dentures	Oral Hygiene	Toothache
Recurrent Tonsillitis (N=54)	1.741	0	0	0.5	0	0.111	0	1.093	0.037
Tonsillar Hypertrophy (N=36)	0.889	0	0	0.25	0	0	0	0.639	0
Peritonsillar Abscess (N=23)	1.609	0	0	0.435	0	0.174	0	1	0
Focal Infection (N=10)	1.1	0	0.1	0.2	0	0	0	0.8	0

Intergroup comparison of OHAT scores

Total Score

The habitual tonsillitis group had a significantly higher total OHAT score compared to the tonsillar hypertrophy group (P = 0.001; Table 2).

**Table 2:** Intergroup Comparison of OHAT Scores (Total Score） *The habitual tonsillitis group had a significantly higher total OHAT score compared to the tonsillar hypertrophy group (P = 0.001） Kruskal–Wallis test with post hoc multiple comparisons. OHAT: Oral Health Assessment Tool

	Standard Error	Standardized Test Statistic	P-value
Tonsillar Hypertrophy VS Focal Infection	12.184	-0.518	0.604
Tonsillar Hypertrophy VS Peritonsillar Abscess	9.098	2.09	0.037
Tonsillar Hypertrophy VS Recurrent Tonsillitis	7.334	3.197	0.001※
Focal Infection VS Peritonsillar Abscess	12.911	0.984	0.325
Focal Infection VS Recurrent Tonsillitis	11.734	1.46	0.144
Peritonsillar Abscess VS Recurrent Tonsillitis	8.487	0.522	0.602

No significant differences were observed between the tonsillar hypertrophy and focal infection groups, the tonsillar hypertrophy and peritonsillar abscess groups, the focal infection and peritonsillar abscess groups, the focal infection and habitual tonsillitis groups, or the peritonsillar abscess and habitual tonsillitis groups.

Gums and Mucosa

No significant differences in OHAT gums and mucosa scores were observed among the four groups: tonsillar hypertrophy, focal infection, peritonsillar abscess, and habitual tonsillitis (Table 3).

**Table 3:** Intergroup Comparison of the OHAT Score (Gums and Mucosa） No significant differences in OHAT gums and mucosa scores. Kruskal–Wallis test with post hoc multiple comparisons. OHAT: Oral Health Assessment Tool

	Standard Error	Standardized Test Statistic	P-value
Tonsillar Hypertrophy VS Focal Infection	10.702	0.28	0.779
Tonsillar Hypertrophy VS Peritonsillar Abscess	11.34	1.098	0.272
Tonsillar Hypertrophy VS Recurrent Tonsillitis	10.307	1.612	0.107
Focal Infection VS Peritonsillar Abscess	7.992	1.183	0.237
Focal Infection VS Recurrent Tonsillitis	6.442	2.113	0.035
Peritonsillar Abscess VS Recurrent Tonsillitis	7.454	0.557	0.577

Remaining Teeth

No significant differences in OHAT remaining teeth scores were observed among the four groups: tonsillar hypertrophy, focal infection, peritonsillar abscess, and habitual tonsillitis (Table 4).

**Table 4:** Intergroup Comparison of OHAT Scores (Remaining Teeth) No significant differences in OHAT remaining teeth scores. Kruskal–Wallis test with post hoc multiple comparisons. OHAT: Oral Health Assessment Tool

	Standard Error	Standardized Test Statistic	P-value
Tonsillar Hypertrophy VS Focal Infection	4.756	0	1
Tonsillar Hypertrophy VS Peritonsillar Abscess	3.552	1.53	0.126
Tonsillar Hypertrophy VS Recurrent Tonsillitis	2.863	1.578	0.114
Focal Infection VS Peritonsillar Abscess	5.04	1.078	0.281
Focal Infection VS Recurrent Tonsillitis	4.581	0.986	0.324
Peritonsillar Abscess VS Recurrent Tonsillitis	3.313	-0.277	0.782

Oral Hygiene

Consistent with the total OHAT score, the habitual tonsillitis group had a significantly higher OHAT oral hygiene score than the tonsillar hypertrophy group (P ＜ 0.001; Table 5).

**Table 5:** Intergroup Comparison of OHAT Scores (Oral Hygiene) *The habitual tonsillitis group had a significantly higher OHAT oral hygiene score than the tonsillar hypertrophy group (P < 0.001) Kruskal–Wallis test with post hoc multiple comparisons. OHAT: Oral Health Assessment Tool

	Standard Error	Standardized Test Statistic	P-value
Tonsillar Hypertrophy VS Focal Infection	11.199	-0.709	0.479
Tonsillar Hypertrophy VS Peritonsillar Abscess	8.363	2.084	0.037
Tonsillar Hypertrophy VS Recurrent Tonsillitis	6.741	3.322	＜ 0.001 ※
Focal Infection VS Peritonsillar Abscess	11.867	0.8	0.424
Focal Infection VS Recurrent Tonsillitis	10.785	1.34	0.18
Peritonsillar Abscess VS Recurrent Tonsillitis	7.8	0.636	0.525

No significant differences were observed between the tonsillar hypertrophy and focal infection groups, the tonsillar hypertrophy and peritonsillar abscess groups, the focal infection and peritonsillar abscess groups, the focal infection and habitual tonsillitis groups, or the peritonsillar abscess and habitual tonsillitis groups.

Other Items

All patients were classified as healthy for the lips, saliva, and dentures. The tongue exhibited changes in one patient in the focal infection group, and toothache exhibited changes in two patients in the habitual tonsillitis group.

## Discussion

Classical tonsillectomy, which involves complete removal of the tonsils under general anaesthesia, is a widely performed surgical procedure for multiple indications [5]. In the United States, over 500,000 tonsillectomies are performed annually on both paediatric and adult patients [6]. Although postoperative outcomes and complications have been well described, no comparative studies have evaluated the association between the preoperative oral environment and surgical indications [7-10]. Previous studies examining the bacterial flora of the tonsils and oral cavity have compared microbial communities within tonsillar tissue between obese and non-obese children. These studies suggest that the oral microbial environment, particularly microbial communities in saliva and the tonsils, is associated with tonsillar hypertrophy and chronic tonsillitis. In addition, the diversity and composition of oral microbiota have been associated with systemic conditions, including obesity and sleep apnea syndrome. These findings suggest that dysbiosis of the oral microbial flora may contribute to tonsillar inflammation and hypertrophy [1]. Treponema species, causative agents of periodontal disease, are present in saliva and the tonsils. As oral hygiene influences the tonsillar microbial environment, improvements in oral hygiene may contribute to the prevention of tonsillitis [2]. In addition, disruption of the oral microbiota may trigger inflammatory diseases, such as periodontal disease and tonsillitis, whereas maintenance of a healthy oral microbiome prevents pathogen colonization and contributes to immune system regulation [3].

In this OHAT-based assessment, significant differences were observed in the total scores and oral hygiene items between the tonsillar hypertrophy and habitual tonsillitis groups. The mean ages were 26 years for the habitual tonsillitis group, 37 years for the peritonsillar abscess group, 36 years for the focal infection group, and 15 years for the tonsillar hypertrophy group, over 10 years younger than the other groups. This age-related difference is likely attributable to oral microbial diversity-assessed by richness, evenness, and the Shannon index, which is significantly higher in children, indicating a more stable oral environment, whereas adults exhibit greater variability [11]. In addition, children are less prone to accumulation of calculus, food debris, and plaque compared to adults.

Furthermore, although previous studies have reported increased Prevotella species in tonsillitis compared to non-tonsillitis mouthwashes, suggesting that inflammation may influence microbial communities, and have noted differing associations between tonsillar microbiota in chronic tonsillitis versus obstructive sleep apnea and hypertrophic tonsils, no significant differences were observed between the groups in our study [12-14]. Possible explanations include resolution of acute inflammation by the time of the preoperative assessment and the OHAT evaluation potentially not fully capturing the inflammatory state.

Our study has several limitations. First, the OHAT is primarily designed as a screening tool for older adults and individuals requiring care, and its sensitivity may be inadequate for assessing younger or otherwise healthy populations [15-17]. Second, the evaluation was based on a single visual observation at a specific time point, without accounting for temporal variations in the oral environment or seasonal influences. Furthermore, the microbiological correlation between the oral cavity and pharynx has not been measured. Future studies should incorporate more detailed oral hygiene metrics and microbiological analyses to investigate the relationship between tonsillar disease and the oral environment from multiple perspectives.

## Conclusions

Evaluation of the intraoral environment prior to palatine tonsillectomy using the OHAT revealed significant differences between the habitual tonsillitis and tonsillar hypertrophy groups. Age-related factors appear to influence these findings, emphasizing the need for future studies to incorporate multifaceted assessments, including the oral microbiome by a bacterial count counter and inflammatory status.

## References

[1] Park J, Lee KE, Choi DH (2023). The association of tonsillar microbiota with biochemical indices based on obesity and tonsillar hypertrophy in children. Sci Rep.

[2] Choi DH, Park J, Choi JK (2020). Association between the microbiomes of tonsil and saliva samples isolated from pediatric patients subjected to tonsillectomy for the treatment of tonsillar hyperplasia. Exp Mol Med.

[3] Kilian M, Chapple IL, Hannig M (2016). The oral microbiome - an update for oral healthcare professionals. Br Dent J.

[4] Chalmers JM, King PL, Spencer AJ, Wright FA, Carter KD (2005). The oral health assessment tool--validity and reliability. Aust Dent J.

[5] Randall DA (2020). Current indications for tonsillectomy and adenoidectomy. J Am Board Fam Med.

[6] Hall MJ﻿, Schwartzman A﻿, Zhang J﻿, Liu X (2017). Ambulatory surgery data from hospitals and ambulatory surgery centers: United States, 2010. Natl Health Stat Report.

[7] Tzelnick S, Hilly O, Vinker S, Bachar G, Mizrachi A (2020). Long-term outcomes of tonsillectomy for recurrent tonsillitis in adults. Laryngoscope.

[8] Schwentner I, Höfer S, Schmutzhard J, Deibl M, Sprinzl GM (2007). Impact of tonsillectomy on quality of life in adults with chronic tonsillitis. Swiss Med Wkly.

[9] Patel SD, Daher GS, Engle L, Zhu J, Slonimsky G (2022). Adult tonsillectomy: an evaluation of indications and complications. Am J Otolaryngol.

[10] Chen MM, Roman SA, Sosa JA, Judson BL (2014). Safety of adult tonsillectomy: a population-level analysis of 5968 patients. JAMA Otolaryngol Head Neck Surg.

[11] Burcham ZM, Garneau NL, Comstock SS, Tucker RM, Knight R, Metcalf JL (2020). Patterns of oral microbiota diversity in adults and children: a crowdsourced population study. Sci Rep.

[12] Yeoh YK, Chan MH, Chen Z (2019). The human oral cavity microbiota composition during acute tonsillitis: a cross-sectional survey. BMC Oral Health.

[13] Chuang HH, Hsu JF, Chuang LP (2021). Different associations between tonsil microbiome, chronic tonsillitis, and intermittent hypoxemia among obstructive sleep apnea children of different weight status: a pilot case-control study. J Pers Med.

[14] Wu S, Hammarstedt-Nordenvall L, Jangard M (2021). Tonsillar microbiota: a cross-sectional study of patients with chronic tonsillitis or tonsillar hypertrophy. mSystems.

[15] Simpelaere IS, Van Nuffelen G, Vanderwegen J, Wouters K, De Bodt M (2016). Oral health screening: feasibility and reliability of the oral health assessment tool as used by speech pathologists. Int Dent J.

[16] Klotz AL, Zajac M, Ehret J, Hassel AJ, Rammelsberg P, Zenthöfer A (2020). Development of a German version of the Oral Health Assessment Tool. Aging Clin Exp Res.

[17] BaHammam FA, McCracken GI, Wassall R, Durham J, Abdulmohsen B (2022). Measurement properties, interpretability and feasibility of instruments measuring oral health and orofacial pain in dependent adults: a systematic review. BMC Oral Health.

